# Artificial Regulation of Water Level and Its Effect on Aquatic Macrophyte Distribution in Taihu Lake

**DOI:** 10.1371/journal.pone.0044836

**Published:** 2012-09-18

**Authors:** Dehua Zhao, Hao Jiang, Ying Cai, Shuqing An

**Affiliations:** Department of Biological Science and Technology, Nanjing University, Nanjing, China; The Australian National University, Australia

## Abstract

Management of water levels for flood control, water quality, and water safety purposes has become a priority for many lakes worldwide. However, the effects of water level management on the distribution and composition of aquatic vegetation has received little attention. Relevant studies have used either limited short-term or discrete long-term data and thus are either narrowly applicable or easily confounded by the effects of other environmental factors. We developed classification tree models using ground surveys combined with 52 remotely sensed images (15–30 m resolution) to map the distributions of two groups of aquatic vegetation in Taihu Lake, China from 1989–2010. Type 1 vegetation included emergent, floating, and floating-leaf plants, whereas Type 2 consisted of submerged vegetation. We sought to identify both inter- and intra-annual dynamics of water level and corresponding dynamics in the aquatic vegetation. Water levels in the ten-year period from 2000–2010 were 0.06–0.21 m lower from July to September (wet season) and 0.22–0.27 m higher from December to March (dry season) than in the 1989–1999 period. Average intra-annual variation (CV_a_) decreased from 10.21% in 1989–1999 to 5.41% in 2000–2010. The areas of both Type 1 and Type 2 vegetation increased substantially in 2000–2010 relative to 1989–1999. Neither annual average water level nor CV_a_ influenced aquatic vegetation area, but water level from January to March had significant positive and negative correlations, respectively, with areas of Type 1 and Type 2 vegetation. Our findings revealed problems with the current management of water levels in Taihu Lake. To restore Taihu Lake to its original state of submerged vegetation dominance, water levels in the dry season should be lowered to better approximate natural conditions and reinstate the high variability (i.e., greater extremes) that was present historically.

## Introduction

Because of the important ecological and socioeconomic functions of aquatic macrophytes, such as stabilization of sediments, regulation of the nutrient cycle, slowing of water currents and fishery maintenance, numerous studies over the past three decades have focused on the dynamics of aquatic macrophytes in freshwater ecosystems and identification of the forces driving their abundances and distributions [1]–[4]. Water quality degradation of the world’s freshwater ecosystems over the past decades has led to extensive decreases in the area occupied by aquatic macrophytes as well as species losses [5], [6]. Promoting the recovery of aquatic macrophytes has become a critical step in the restoration and rehabilitation of these degraded aquatic ecosystems [7]–[9].

Water levels, which are controlled by both natural conditions (e.g., meteorological and catchment characteristics) and local human activities (e.g., flood-control projects and artificial water transfer) [10], have been thought to be responsible for the variability in biomass and species composition of aquatic macrophytes in many freshwater ecosystems of the world [10]–[16]. Although artificial management and manipulation of water levels have been practiced widely, the effect of managed water levels on aquatic macrophytes has not been fully understood in most cases because of the complex relationship between macrophytes and water level [10], [14], [17], [18].

Taihu Lake is the third-largest freshwater lake in China, occupying a surface area of 2,425 km^2^ [19]–[21]. Due to rapid industrialization and urbanization, nutrient concentrations have increased continually during the past decades, and eutrophication has become a dominant water quality problem [22]. In an effort to recover the degraded aquatic ecosystem of Taihu Lake, numerous costly water conservation projects have been implemented in recent years. Planting and restoration of aquatic macrophytes for the purpose of removing excess nutrients are key facets of most of these projects [8], [23], [24].

Meanwhile, large amounts of water have been flushed into the lake from the Yangtze River since 2001 under the premise of “conquering the unmoving with the moving, diluting the polluted with the clean, supplementing low flow with ample flow” to improve water quality and control algal blooms [25], [26]. Following the notorious blue-green algal bloom that occurred in the summer of 2007 and which resulted in serious drinking water shortages in Wuxi City [27], [28], one of the most economically developed cities in Jiangsu Province, even more water was pumped into the lake [19]. Concurrently, more than 28,000 km of sea walls, river banks, embankments and polder dikes were built to control flooding [19]. As a result, water levels and their dynamics, especially intra-annual dynamics, have changed substantially in Taihu Lake.

Despite the considerable changes in the water levels in Taihu Lake, little attention has been focused on the effects on aquatic macrophytes, even though inter- and intra-annual water levels have been identified as one of the most important forces driving variability in aquatic macrophyte distribution [10], [14]. Because aquatic macrophytes are distributed over such a large area (i.e. hundreds of square kilometers) [22], slight variations in water levels are likely to have precipitated a decrease or increase in the distribution of the macrophytes on the scale of tens of kilometers, greatly affecting their combined ability to remove nutrients and act as a source of food for fisheries [29], [30]. Many water conservation projects focusing on planting aquatic macrophytes have been conducted [23], [24], [31], but it is likely more economical to protect and restore the existing communities of aquatic macrophytes. Protection and restoration, however, requires that increased attention be focused on understanding the effects of inter- and intra-annual water levels on aquatic macrophytes in the lake.

Although some authors have found correlations between the variation in aquatic vegetation and water levels in regard to aquatic systems at large temporal scales, most of those studies were based on either limited short-term or discrete long-term data [12], [13], [32], and thus the results are either narrowly applicable or easily confounded by the cumulative effect of other environmental factors with gradual temporal variation, such as trophic status. In this project, we mapped aquatic vegetation distribution between 1989 and 2010 based on remote sensing images with spatial resolutions from 15 to 30 m. Our objective was to determine the effect of managed water level on the distribution and composition of aquatic macrophytes in Taihu Lake.

## Materials and Methods

### 2.1 Ethics Statement

No specific permits were required for the described field studies. The location studied is not privately-owned or protected in any way, and the field studies did not involve endangered or protected species.

### 2.2 Study Area

The Taihu Lake catchment plays an important role in China’s political economy, containing 3.7% of the country’s population, creating 11.6% of Chinese gross domestic product (GDP) and contributing 19% of total revenue while comprising only 0.4% of the land area of China [19]. Since the 1950s and especially since the 1980s, human activities have placed increased pressure on the lake’s ecological components [21]. Our study area was limited to the areas identified in the remote sensing images as being covered by water in winter, the season when water levels were lowest and the topsoil of most patches of emergent vegetation was dry. As a result, most emergent vegetation was excluded from this work. We chose to exclude most emergent vegetation in order to reduce the effects of human activities on the relationship between aquatic vegetation and water levels since human activities have drastically altered the emergent vegetation of Taihu Lake through large-scale construction of embankments and buildings, as well as vegetation restoration or destruction [31].

Pen-fishing has also had a large influence on the distribution of aquatic vegetation due to farmers’ activities such as planting and harvest. The area subject to pen-fishing has varied dramatically over the past two decades, i.e. a gradual increase between 1990 and 2005 followed by a sudden decrease after 2007, when an extensive blue-green algal bloom occurred and resulted in serious drinking water shortages in Wuxi City [27], [28]. However, pen-fishing activities have been limited primarily to the East Bay of Taihu Lake [31], [33]. Therefore, the East Bay was also excluded from this study to minimize the confounding influence of pen-fishing on aquatic macrophyte distribution.

From 1960 to 2000, human activities resulted in a worsening of the water quality of Taihu Lake at an approximate rate of one grade every 10–15 years [23]. To improve water quality, much effort has been expended on lake restoration, especially after 2000. Artificial management of water levels through pumping of water as well as construction of embankments and dams was a common strategy for improving water quality and controlling blue-green algal bloom. In particular, increased amounts of water were pumped into Taihu Lake from the Yangtze River after 2000 [19], [25]. Therefore, our study period ranged from 1989 to 2010 to encompass the ten years before and after 2000, i.e., Period 1 (1989–1999) and Period 2 (2000–2010). However, high-quality remote sensing images were available for only sixteen of the years between 1989 and 2010.

### 2.3 Field Surveys

The aquatic vegetation of Taihu Lake was grouped into four types: emergent, floating-leaf, floating and submerged [22], [31]. Dominant species in the lake included *Phragmites communis*, *Zizania latifolia*, *Nymphoides peltatum*, *Trapa natans*, *Potamogeton malaianus* and *Vallisneria spiralis*, as identified by field observations as well as previously published studies [30], [31]. We divided the aquatic vegetation into two types according to their spectral characteristics. Type 1 represented the typical green vegetation identified in remote sensing images by lower red band reflectance paired with higher near-infrared band reflectance than other ground cover types and included emergent, floating-leaf and floating vegetation having some green leaves above the water surface. As previously noted, most emergent vegetation was excluded from Type 1. Type 2 consisted of the submerged vegetation, which had all green leaves submerged beneath the water surface, thus distinguishing it from typical green vegetation in remote sensing images. Because Type 1 vegetation had a higher signal intensity than Type 2, areas containing both Type 1 and Type 2 vegetation were classified as emergent vegetation.

**Figure 1:**
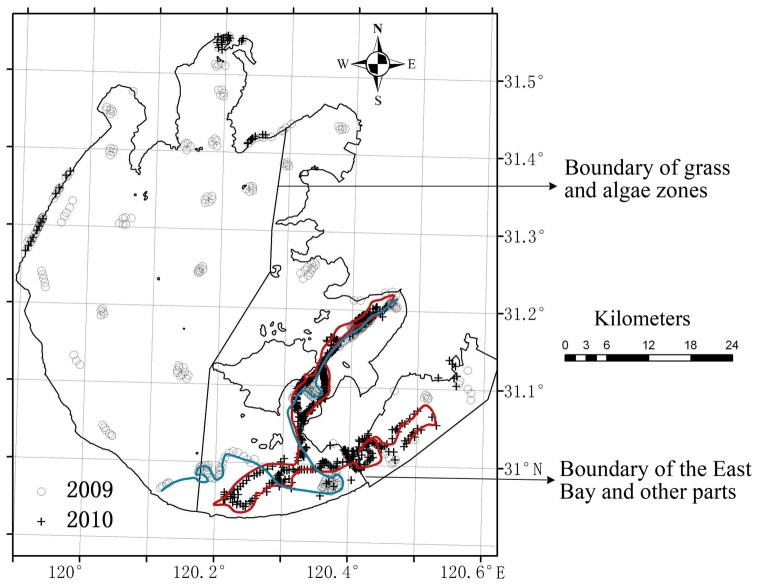
The study area showing the distribution of 965 ground truth samples (426 in 2009 and 539 in 2010) in Taihu Lake, China.

To obtain data for developing and validating models to identify aquatic vegetation, we conducted field surveys on 14–15 September 2009 and 27 September 2010. A total of 783 samples were collected in open water or aquatic vegetation (mostly floating-leaf or submerged) of Taihu Lake, including the East Bay. An additional 182 samples of reed (emergent) vegetation or terrestrial areas (e.g., shoreline roads and buildings such as docks, businesses and factories) were obtained from a 1∶50,000 land use and land cover map due to logistical difficulties in maneuvering a boat in the dense reed vegetation. A total of 426 and 539 ground truth samples were collected in 2009 and 2010, respectively (Fig. 1). At each field sampling plot, photographs were taken using a digital camera (IXUS 950, Canon) held at about 1.2 m above the water surface, with the camera axis angled about 30 degrees down from the horizon. The position of each photograph was geo-located using a portable GPS receiver with an accuracy of 3 m. In the laboratory, all the photographs were interpreted visually and classified as Type 1, Type 2 or open water sediment.

### 2.4 Image Processing

Multispectral TM, ETM+, SPOT-4, HJ, and CBERS remote sensing images were used in this study, with spatial resolutions ranging from 15 to 30 meters. Following the recommended standard for aquatic remote sensing [34], we selected images containing no more than 10% cloud cover at the study area. The cosine approximation model (COST; Chavez, 1996), which has been implemented successfully in other aquatic remote sensing studies [35], [36], was used to apply atmospheric corrections to all the images used. Prior to atmospheric correction, cloud-contaminated pixels were removed from all images using interactive interpretation. Geometric correction was applied using second-order polynomials with accuracy higher than 0.5 pixel.

**Table 1:** Remote sensing images used in this study with associated dates.

Years	Image-1	Image-2	Image-3	Image-4
1989	TM, 1/14	TM, 7/17	TM, 10/21	
1991	TM, 1/12	TM, 7/23	TM, 8/24	
1992	TM, 2/16	TM, 7/25	TM, 8/10	
1995	TM, 2/24	TM, 8/3	TM, 8/19	
1996	TM, 1/10	TM, 7/20	TM, 9/6	
1998	TM, 1/31	TM, 8/11	TM, 7/10	
2000	ETM+, 3/18	ETM+, 8/8	CBERS, 9/16	ETM+, 10/12
2001	ETM+, 1/15	ETM+, 7/26	ETM+, 9/28	
2002	ETM+, 2/3	ETM+, 7/13	TM, 8/22	
2003	ETM+, 2/6	ETM+, 8/1	SPOT, 8/23	
2004	ETM+, 2/9	ETM+, 8/3	CBERS, 8/8	ETM+, 8/19
2005	ETM+, 3/31	ETM+, 6/19	ETM+, 9/7	
2006	ETM+, 3/2	CBERS, 8/6	TM, 9/18	ETM+, 9/28
2008	ETM+, 2/20	CBERS, 7/24	ETM+, 8/14	HJ, 9/23
2009	ETM+, 1/13	ETM+, 8/25	HJ, 9/10	
2010	ETM+, 3/13	ETM+, 8/20	ETM+, 9/21	

Because they contain information on seasonal dynamics of both aquatic vegetation and related environmental factors, multiple intra-annual remote sensing images can provide higher accuracy for the identification of aquatic vegetation than a single image [37], [38]. Therefore, we used combinations of winter (between January and March when the biomass of aquatic vegetation was lowest) and summer (between June and October when the biomass of aquatic vegetation was highest) images from each study year between 1989 and 2010. For each study year, at least three clear Landsat images were selected (one from winter and the others from summer) and formed into at least two pairs in which the winter image was paired with each summer image. A total of 36 pairs were used in this study (Table 1).

Aquatic vegetation was mapped using each image combination, so at least two vegetation maps were obtained for each year. Maps for the same year were superimposed and combined according to the following rules: (1) In the grass type zone of Taihu Lake (i.e. the eastern portion in Fig. 1), if a pixel was classified as aquatic vegetation in either map within a single study year, it was classified as aquatic vegetation in the final map. This rule was established primarily because human activities such as harvesting of aquatic vegetation might decrease the distribution of aquatic vegetation, and because particulate matter that is suspended very high in the water column might obscure the submerged vegetation, resulting in underestimation of submerged vegetation [22] at some time during the growing season. (2) In the algae type zone (i.e. the remaining portions of the lake not in the grass type zone in Fig. 1), a pixel was regarded as aquatic vegetation only when it was classified as aquatic vegetation in all maps for a single study year. This rule aimed to reduce classification interference from algal blooms, which occur frequently between May and October [27], [28], [39] and is based on the probability being much lower that algal blooms will appear twice in the same location than that aquatic macrophytes will. The ground truth samples from 2009 and 2010 were used to evaluate the accuracy of the final aquatic vegetation classifications derived from the 2009 and 2010 image pairs.

### 2.5 Analytical Methods

#### 2.5.1 Identification of aquatic vegetation in remote sensing images

**Figure 2:**
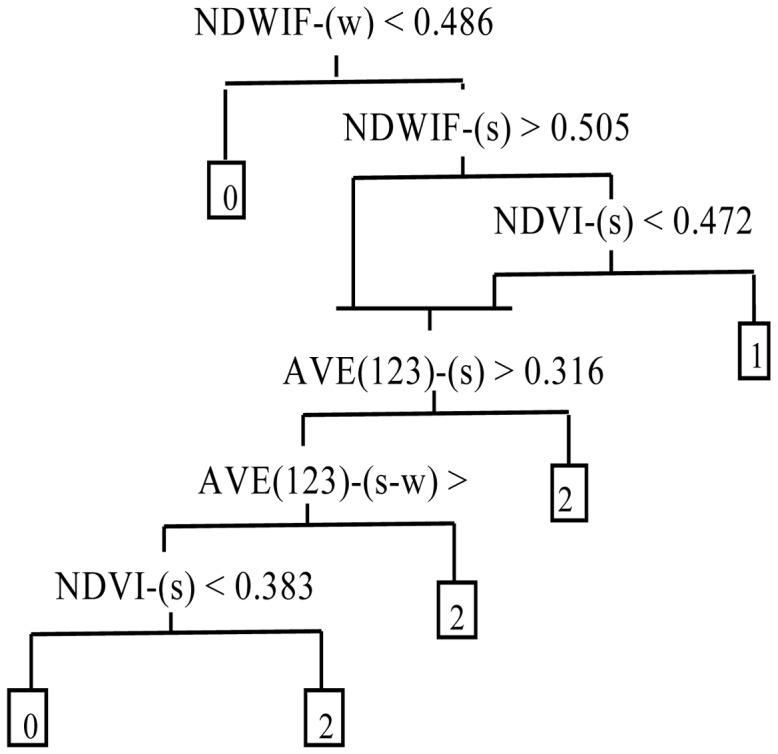
Classification tree models established for Type 1 and Type 2 aquatic vegetation. The numbers 1 and 2 in the end nodes of the classification trees represent Type 1 and Type 2 vegetation, respectively, whereas 0 represents other types. Variables used are the Modified Normalized Difference Water Index (MNDWI), the Normalized Difference Vegetation Index (NDVI) and the average reflectance of the blue, green and red image bands (AVE123). Variables were calculated by season (s  =  summer, w  =  winter) or differences among seasonal values (e.g., s-w).

Classification tree (CT) analysis, which uses recursive dichotomous partitioning of the data according to calculated thresholds, has been used successfully for the identification of aquatic vegetation because of its flexibility with regard to the inclusion of data from multiple sources and of multiple types, such as spectral signals, environmental variables and other variables related to aquatic vegetation growth [38], [40]–[44]. Zhao et al. (2012) developed an improved CT modeling algorithm for identifying emergent, floating-leaf and submerged vegetation from remote sensing images both from different times [45] and from different sensor (Zhao et al. A method for application of classification tree models to map aquatic vegetation using remotely sensed images from different sensors and dates. Sensors, Re-submitted after revision). Because we divided the aquatic vegetation into only two types in this study (i.e. Type 1 and Type 2), minor modifications were made to the CT model structure (Fig. 2).

We obtained the quantitative thresholds for the CT base model structure and thus the final CT models by applying CT analysis to the 2009 image pairs (Fig. 2), attaining an overall classification accuracy of 94.0%, with classification accuracies of 95.6% and 88.8% for Type 1 and Type 2 vegetation, respectively. When the CT models were applied to the image pairs of 2010, overall accuracy was 93.3%, with classification accuracies of 94.2% and 87.9% for Type 1 and Type 2 vegetation, respectively (Table 2). These results suggested that our CT model could be used to effectively identify the aquatic vegetation in Taihu Lake. Therefore, we used the models to map the distribution of aquatic vegetation in Taihu Lake from 1989 to 2010.

**Table 2:** Confusion matrix of the CT models developed in this paper as applied to 2009 and 2010 data, respectively (in number of field samples).

			Prediction				
			Type 1	Type 2	Other types	Classificationaccuracy (%)	Overall accuracy (%)
2009	Truth	**Type 1**	130	4	2	95.6	94.0
		**Type 2**	5	103	8	88.8	
		**Other types**	1	4	145	96.7	
2010	Truth	**Type 1**	175	7	4	94.1	93.3
		**Type 2**	6	102	8	87.9	
		**Other types**	0	8	186	95.9	

#### 2.5.2 Evaluation of water level effects on aquatic vegetation

The annual Coefficient of Variation (CV_a_) was calculated to describe the inter-annual fluctuation of water levels:
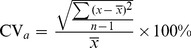
(1)


where x and 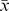 are average monthly water level and average annual water level, respectively.

We used regression analysis to investigate the effects of water level variation and CV_a_ fluctuation on the distribution of aquatic vegetation through time. However, using un-transformed values for water level and area of aquatic vegetation is unlikely to reflect the true relationship because of the inevitable temporal autocorrelation of aquatic vegetation as well as the confounding influence of gradual changes in environmental factors such as water nutrient content, chemical oxygen demand (COD) and water clarity. Therefore, we transformed the water level and aquatic vegetation area variables using the variability from one year to the next before performing the regression analysis. This was accomplished by subtracting the previous year values from the focus year values using the same time period. Thus, water level variability was calculated as:
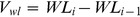
(2)


Where WL_i_ and WL_i−1_ are the average water levels or CV_a_ in the focus year (i) and the year previous to the focus year (i−1), respectively. However, if data for the previous year were missing, data from two years prior to the focus year (i−2), or three years prior (i−3) if data were also missing for i−2, were used instead. Variability in aquatic vegetation area was calculated as:
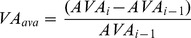
(3)


Where AVA_i_ and AVA_i−1_ are aquatic vegetation areas in the focus year (i) and year previous to the focus year (i−1), respectively. Similar to Equation (2), if aquatic vegetation data were not available for the previous year, it was replaced by the data for the closest year for which data were available.

Because most of the images used to map aquatic vegetation distribution were dated prior to October with only two exceptions (Table 1), water levels between October and December in a certain year did not influence aquatic vegetation area of the same year. However, October-December water levels probably influenced the aquatic vegetation of the following year. Therefore, October through December water levels of the year previous to the focus year were used to analyze relationships between monthly water levels and aquatic vegetation.

## Results

### 3.1 Temporal Dynamics of Water Level

**Figure 3:**
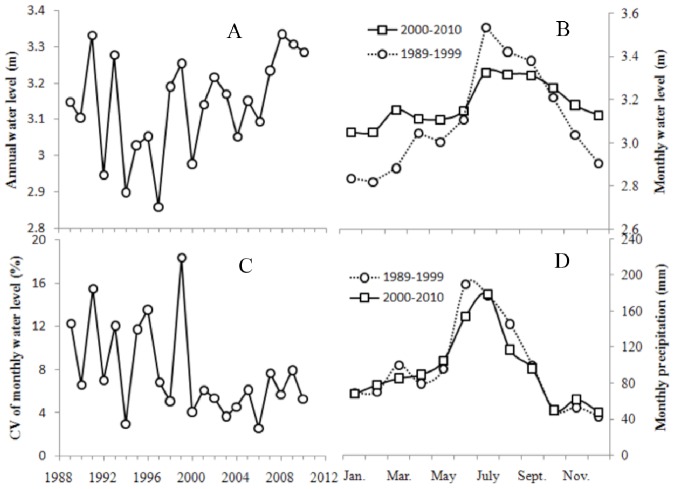
Inter- and intra- annual dynamics of water level and precipitation in Taihu Lake. (A) annual water level dynamics between 1989 and 2010; (B) average monthly water levels for the two 10-year time periods examined in this study; (C) intra-annual fluctuation of water levels (CV_a_) from 1989–2010; and (D) average monthly precipitation for the two 10-year time periods examined in this study.

Between 1989 and 2010, annual average water levels fluctuated between 2.86 m and 3.33 m, with no significant inter-annual trend (Fig. 3A). The average water level in Period 2 (2000–2010) was 3.18 m, slightly higher than that in Period 1 (i.e. 1989–1999, average = 3.10 m). However, substantially different intra-annual dynamics were observed between the two temporal periods (Fig. 3B), with more stable water levels in Period 2 than in Period 1. In Period 1, monthly water levels ranged from 2.57 m to 4.61 m with the annual Coefficient of Variation (CV_a_) ranging from 3.06% (1994) to 18.41% (1999) and averaging 10.21%. However, in Period 2, monthly water levels ranged from 2.76 m (2006) to 3.98 m (2009), with CV_a_ ranging from 2.65% to 7.94% and averaging 5.41% (Fig. 3C). CV_a_ in Period 1 was significantly higher than that in Period 2 (*p* = 0.01). For July, August and September, monthly water levels in Period 2 were 0.064 to 0.21 m lower than those in Period 1, whereas monthly water levels in Period 2 for the remaining months were 0.042 to 0.27 m higher than those in Period 1. Thus, our results indicate differences in intra-annual dynamics of water level between Period 1 and Period 2.

Monthly precipitation in Period 1 was slightly higher (1.49 to 30.48 mm) than that in Period 2 for January, March, June, July, August and September; for the other six months, monthly precipitation in Period 1 was slightly lower (0.03 to 9.80 mm) than that in Period 2 (Fig. 3D). Annual precipitation in Period 1 (1175.4 mm) was slightly higher than that in Period 2 (1132.1 mm). Thus, precipitation and water level showed different intra-annual variation patterns between the two periods. These results suggested that climatic conditions were not responsible for the greater stability of intra-annual water level in Period 2 than in Period 1.

### 3.2 Temporal Dynamics of Aquatic Vegetation Distribution Area

**Figure 4:**
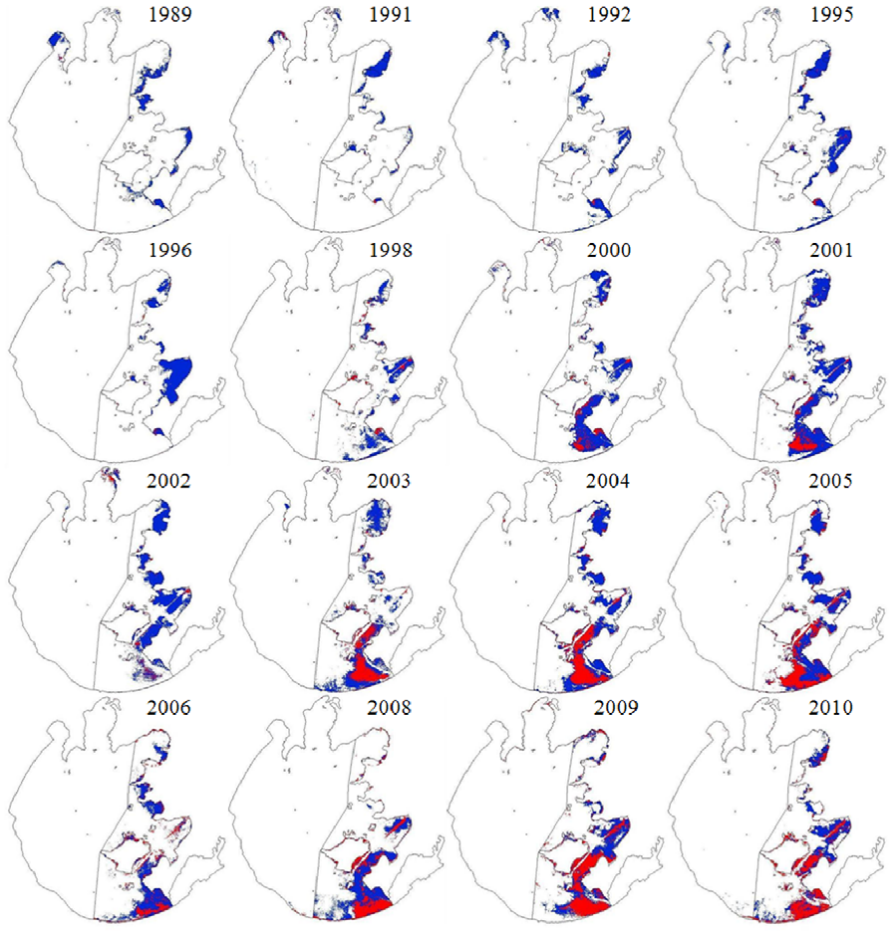
Distribution of aquatic vegetation of Type 1 (red) and Type 2 (blue) between 1989 and 2010. Type 1 vegetation consisted of emergent, floating-leaf and floating vegetation, whereas Type 2 consisted of submerged vegetation.

**Figure 5:**
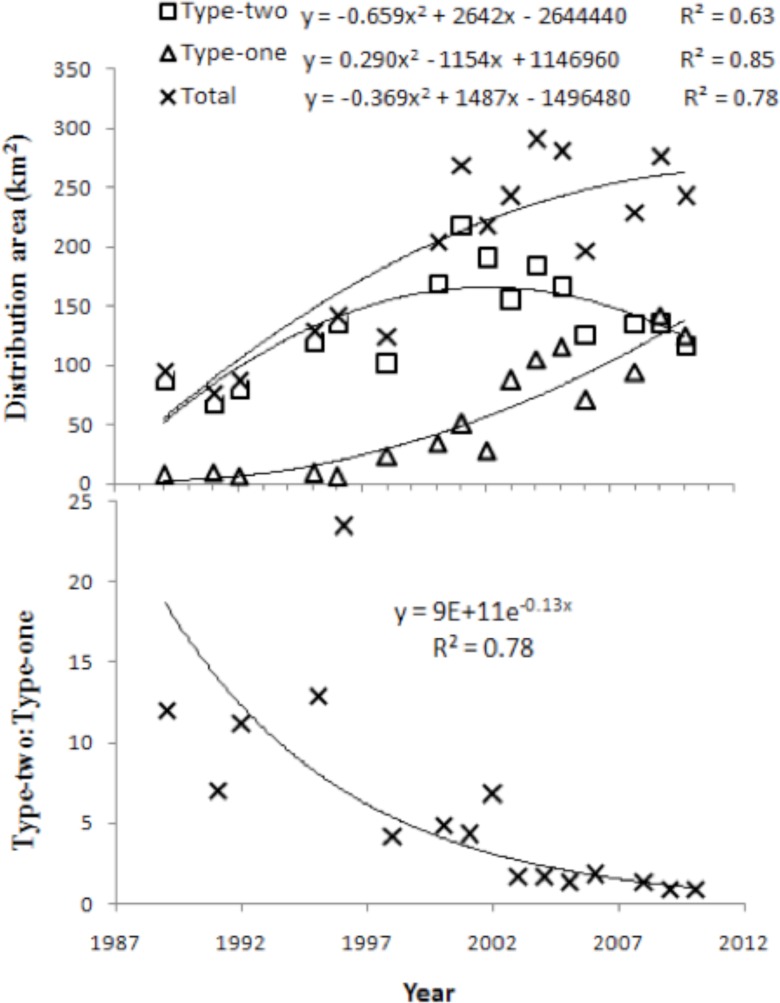
Temporal trends (1989–2010) of the distribution area of aquatic vegetation components and derivatives. Type 1 vegetation consisted of emergent, floating-leaf and floating vegetation, whereas Type 2 consisted of submerged vegetation.

From 1989 to 2010, aquatic vegetation was distributed primarily in the eastern part of the lake (Fig. 4). The spatial pattern of distribution experienced some changes, with aquatic vegetation shifting gradually from the northeast to the southeast. Substantial changes were observed in both distribution area and composition of aquatic vegetation during the study period (Fig. 5). The area covered by Type 1 vegetation ranged from 5.80 km^2^ (1996) to 142.5 km^2^ (2009), with an average of 57.3 km^2^, whereas Type 2 vegetation covered an area ranging from 68.3 km^2^ (1991) to 190.8 km^2^ (2001), with an average of 137.4 km^2^. The total aquatic vegetation area (i.e., the sum of Type 1 and Type 2 vegetation) ranged from 77.9 km^2^ (1991) to 282.0 km^2^ (2005), with an average of 194.7 km^2^, and the ratio of Type 2 to Type 1 vegetation ranged from 0.94 (2010) to 23.4 (1996), with an average of 6.07. Significant temporal dynamics were observed for each variable from 1989 to 2010 (*p*  = 0.01). Both total aquatic vegetation area and area of Type 1 vegetation increased significantly over the study period (*p*  = 0.01). Area of Type 2 vegetation increased before 2001 and then decreased (*p*  = 0.01), and the ratio of Type 2 to Type 1 vegetation decreased steadily over the study period (*p*  = 0.01).

The area covered by aquatic vegetation also differed between the two inter-annual temporal periods examined. Average Type 2 vegetation area increased from 99.2 km^2^ in Period 1 to 160.4 km^2^ in Period 2, an increase of 61.6%, and average Type 1 vegetation area increased 7.14-fold, from 10.5 km^2^ in Period 1 to 85.4 km^2^ in Period 2. Total vegetation area increased 124.0%, from 109.7 km^2^ in Period 1 to 245.8 km^2^ in Period 2. Finally, the ratio of Type 2 to Type 1 vegetation decreased from 11.8 in Period 1 to 2.62 in Period 2.

### 3.3 Relationship between Water Level and Aquatic Vegetation Area

Firstly, we investigated whether the annual average water levels and intra-annual fluctuation of water levels (CV_a_) influenced inter-annual aquatic vegetation area and its derivations. No significant correlations were found between V_wl_ and VA_ava_ using either annual averages or CV_a_ for either vegetation type, total vegetation area, or the ratio of Type 2 to Type 1 vegetation (Table 3).

**Table 3:** Linear correlation coefficients between variation of aquatic vegetation (VA_ava_) and variation of water level (V_wl_) (n = 15).

Aquatic Vegetation Parameter	Annual average	CV_a_
Type 2	−0.33	0.29
Type 1	0.21	−0.34
Total	−0.25	0.33
Type 2:Type 1	−0.40	0.02

Type 1 vegetation consisted of emergent, floating-leaf and floating vegetation, whereas Type 2 consisted of submerged vegetation.

**Figure 6:**
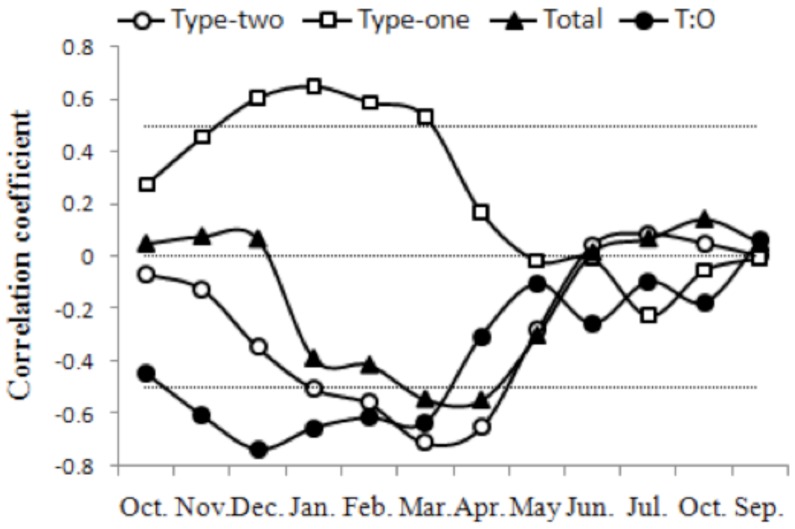
Linear correlation coefficients between monthly water level (in V_wl_) and the distribution area of aquatic vegetation components (in VA_ava_) during the time period 1989–2010. Type 1 vegetation consisted of emergent, floating-leaf and floating vegetation, whereas Type 2 consisted of submerged vegetation. T:O is the ratio of Type 2 to Type 1 vegetation area.

Secondly, we tested the correlations between monthly average water levels and aquatic vegetation (Fig. 6). Significant positive correlations were found between Type 1 vegetation area and monthly water levels from December to March (*p*  = 0.05), whereas significant negative correlations were found between Type 2 vegetation area and monthly water levels from January to April (*p*  = 0.05). Total vegetation area was negatively correlated with monthly water levels from March to April (*p*  = 0.05), and the ratio of Type 2 to Type 1 vegetation was negatively correlated with monthly water levels from November to March (*p*  = 0.05). These results suggested that water levels in late winter and early spring (traditional dry season) significantly influenced aquatic vegetation area.

## Discussion

### 4.1 Temporal Changes

We found significant relationships between water level from December to March and the area of Type 1 vegetation, with increases in Type 1 vegetation occurring steadily from 1989 to 2010 (and consequently for the 2000–2010 period relative to the 1989–1999 period) and coinciding with increased water levels during the dry season. The aquatic macrophyte communities in very shallow lakes such as Taihu Lake [19] may be especially sensitive to variation in water levels. In addition, according to the stable state theory proposed by Scheffer et al. (2003) [46], nutrient enrichment should shift the vegetation in freshwater systems toward dominance by floating plants; in fact, nutrient concentrations, as well as area of Type 1 vegetation, have shown a gradual increase in Taihu Lake over the past 20 years [23], [47]. Therefore, nutrient enrichment and higher dry-season water levels may represent two of the most important factors responsible for the temporal shifts in Type 1 vegetation distribution observed in this study.

We found significant negative relationships between Type 2 vegetation and dry-season monthly water level as well as slightly higher dry-season water levels in 2000–2010 than in 1989–1999. Therefore, water level couldn’t explain the 61.6% increase of Type 2 in 2000–2010 over that in 1989–1999. According to an alternative equilibrium theory [48], [49], submerged vegetation responds in a non-linear way to eutrophication, first increasing then decreasing. Our findings support this theory, with nutrient enrichment increasing over the past 20 years in Taihu Lake and Type 2 vegetation increasing in area until about 2002 and decreasing thereafter. Nutrient enrichment is thought to be responsible for the expansion of aquatic vegetation in numerous lakes throughout the world and probably acted as one of most important driving factors of vegetation dynamics in our study [50], [51]. We speculate that water level probably acted as the dominant factor determining Type 2 vegetation area on a one- to two-year temporal scale, whereas gradual changes in eutrophication probably acted as the dominant driving factor on a longer (∼10 years) temporal scale.

The methodology used for identifying the effect of water level on aquatic vegetation can have a large influence on the results obtained. Despite a few successful reports [10], [11], using non-transformed values of water level and aquatic vegetation area in regression models is unlikely to reveal the actual temporal relationship between the two variables in periods when confounding factors such as nutrient concentration vary considerably. Using transformed parameters such as the V_wl_ and VA_ava_ variables we used in this study instead of non-transformed water level and aquatic vegetation area values has two advantages: (1) it can alleviate the problem of temporal autocorrelation in aquatic vegetation area and its derivatives; and (2) it can help distinguish influences of water level from gradually and continuously increasing or decreasing factors such as water eutrophication [23].

### 4.2 Mechanisms for the Influence of Water Level on Aquatic Vegetation

Underwater light availability, which is affected strongly by water level, is an important mechanism influencing aquatic vegetation. Decreases in available light have been found to be of the most important factors resulting in species disappearance and biodiversity loss both for aquatic and terrestrial vegetation [52], [53]. Many studies have found underwater light to be closely correlated with water level [4], [10], [13], [16], especially for turbid waters such as Taihu Lake [25], [54]. Generally, increases in water level will reduce underwater light availability, especially at the bottom. If underwater light decreases below the threshold of a species’ minimum light requirements, the species will disappear from the community.

Changes in light availability resulting from water level variability can easily explain the negative correlation found between area of Type 2 vegetation, which is submerged, and water level in traditional annual dry seasons. However, the positive correlation between Type 1 vegetation area and water level during the dry season cannot be explained as a direct result of light availability because of the positive effect of light availability on aquatic vegetation growth [55]. A more plausible explanation of the positive correlation would be due to competitive interactions between Type 1 and Type 2 vegetation [56]. According to observations from our field campaign, Type 1 species such as *Nymphoides peltatum* and Type 2 species such as *Potamogeton malaianus* grew widely in mixed communities in Taihu Lake [30], promoting strong competition for space and light. Because Type 2 vegetation is more sensitive to underwater light restrictions, which can be exacerbated by increases in water level, than Type 1 vegetation [55], [57], [58], water level increases can strengthen competitive ability of Type 1 vegetation by inhibiting the growth of Type 2 vegetation, ultimately resulting in the correlation patterns observed.

Our results indicated that water level influenced aquatic vegetation in dry seasons more so than in rainy seasons. This may be a consequence of the phenology of aquatic plants, which are most sensitive to light conditions in the germination and initial growth stages [59]. Most species in Taihu Lake survive winter with tuber-like buds in silt and germinate in the early spring [30], [31]. Water level can directly influence the germination of buds by altering light availability at the lake bottom. Upon entering the rapid growth period, *Nymphoides peltatum* and *Potamogeton malaianus*, two of the most widely distributed species, become less sensitive to water level variability because of their strong morphological plasticity [60]. Our results were consistent with Blindow et al. (1993) and Paillisson and Marion (2006) [11], [49], who found that spring water level influenced the growth of aquatic vegetation.

### 4.3 Management Implications

Our results suggest that regulation of the distribution of aquatic vegetation is feasible through management of water levels in Taihu Lake. Since 2000, and especially since 2007 when a severe blue-green algal bloom resulted in serious drinking water shortages in Wuxi City [27], [28], multiple costly water conservation projects have been conducted in Taihu Lake. Restoration of aquatic vegetation through artificial planting or other means has been one of most common approaches [31] because of the purification function of aquatic vegetation. The significantly better water quality in the eastern coastal area of Taihu Lake relative to other parts of the lake is a solid example of this purification function [60]. Our results indicate that decreasing water levels in the dry season could increase the area occupied by aquatic vegetation in tens of square kilometers, which is a difficult goal to achieve using other restoration strategies such as direct planting.

In addition to regulation of the area occupied by aquatic vegetation generally, our results suggest that artificial control of distinct aquatic vegetation components is possible by regulating water levels. This finding is potentially very useful for lake management because of the different ecological and socioeconomic functions performed by the different aquatic vegetation types such as submerged *vs.* floating-leaf vegetation [29], [30]. For example, submerged vegetation is one of the most important food resources for breeding crabs, which represent an annual harvest value from Taihu Lake of more than two hundred million dollars [29]. Farmers usually must plant submerged vegetation in the lake to act as a food source for the crabs, but water level regulation may represent a more economical and effective alternative. Additionally, decreasing water levels in the dry season will bring intra-annual water level fluctuations closer to natural conditions (i.e. large intra-annual fluctuations [14]) while restoring Taihu Lake to its original state of dominance by submerged vegetation.

Finally, our results suggest that regulation of water levels could be used to better control algal blooms in the lake. One of the main objectives of the current water level regulation strategies, such as the flushing of water into the lake from the Yangtze River, is to control algal blooms [19], [25], [26]. Because algal blooms usually occur between May and October in Taihu Lake, decreases in water level between late winter and early spring will not reduce the effectiveness of the current strategy for controlling algal blooms. On the contrary, careful reductions in water level between late winter and early spring are beneficial to the control of algal blooms in the lake because of their ability to increase the distribution of aquatic vegetation, which will in turn reduce nutrient levels.
